# Stromal Response to Prostate Cancer: Nanotechnology-Based Detection of Thioredoxin-Interacting Protein Partners Distinguishes Prostate Cancer Associated Stroma from That of Benign Prostatic Hyperplasia

**DOI:** 10.1371/journal.pone.0060562

**Published:** 2013-06-06

**Authors:** Elizabeth Singer, Jennifer Linehan, Gail Babilonia, S. Ashraf Imam, David Smith, Sofia Loera, Timothy Wilson, Steven Smith

**Affiliations:** 1 City of Hope, Duarte, California, United States of America; 2 Huntington Medical Research Institute, Pasadena, California, United States of America; UCLA, United States of America

## Abstract

Histological staining of reactive stroma has been shown to be a predictor of biochemical recurrence in prostate cancer, however, molecular markers of the stromal response to prostate cancer have not yet been fully delineated. The objective of this study was to determine whether or not the stromal biomarkers detected with a thioredoxin-targeted nanodevice could be used to distinguish the stroma associated with benign prostatic hyperplasia from that associated with PCA. In this regard, we recently demonstrated that a thioredoxin-targeted nanodevice selectively binds to reactive stroma in frozen prostate tumor tissue sections. To accomplish this, random frozen prostate tissue sections from each of 35 patients who underwent resection were incubated with the nanodevice and graded for fluorescent intensity. An adjacent section from each case was stained with Hematoxylin & Eosin to confirm the diagnosis. Select cases were stained with Masson's Trichrome or immunohistochemically using antibodies to thioredoxin reductase 1, thioredoxin reductase 2 or peroxiredoxin 1. Our results demonstrate that the graded intensity of nanodevice binding to the stroma associated with PCA was significantly higher (p = 0.0127) than that of benign prostatic hyperplasia using the t-test. Immunohistochemical staining of adjacent sections in representative cases showed that none of the two commonly studied thioredoxin interacting protein partners mirrored the fluorescence pattern seen with the nanodevice. However, thioredoxin reductase 2 protein was clearly shown to be a biomarker of prostate cancer-associated reactive stroma whose presence distinguishes the stroma associated with benign prostatic hyperplasia from that associated with prostate cancer. We conclude that the signal detected by the nanodevice, in contrast to individual targets detected with antibodies used in this study, originates from multiple thioredoxin interacting protein partners that distinguish the M2 neutrophil and macrophage associated inflammatory response in prostate cancer-associated stroma from the CD4+ T-Lymphocyte linked inflammation in benign prostatic hyperplasia.

## Introduction

In considering the role of inflammation in prostate cancer, one of the confounding observations is that chronic immune inflammation appears to play a crucial role in both Prostate Cancer (PCA) [1] and Benign Prostatic Hyperplasia (BPH) [2]. BPH is clearly a late-onset phenomenon, and results from the PCPT trial strongly suggest that a diagnosis of BPH is not associated with elevated prostate cancer risk [3], however, a recent report suggests that hospitalization and surgery for BPH can increase the risk of prostate cancer specific death by as much as 8 fold [4]. Here, the wounding associated with surgery is consistent with the hypothesis that a wound response (*i.e*. respiratory-burst type inflammation) is associated with the genesis of aggressive prostate cancers.

Close examination of the type of inflammation associated with BPH suggests that it consists largely of IL-15 and γ-interferon recruited CD4+ T-Lymphocytes [2]. This contrasts with the tumor-associated macrophages present in the tumor microenvironment. In general these macrophages are thought to gradually switch from an M1 to an M2 phenotype during tumor progression, leaving an M2-like phenotype [1] that still produces reactive nitrogen (RNS) [5] and reactive oxygen species (ROS) [1].

For these reasons, stromal cells in the tumor-associated microenvironment are expected to experience exposure to both ROS and RNS. Thioredoxin, a small redox protein, could be involved in the stromal response to this exposure. Thioredoxin expression appears to be a link between oxidative stress and inflammation in that it is a chemoattractant for neutrophils, monocytes and T-cells [6]. Thioredoxin interacting protein partners, like TXNRD2 which reduces H_2_O_2_ to H_2_O [7], and TXNRD1 which appears to be rate limiting in the removal of S-nitrosylated cysteine residues from caspase-3 [8] and perhaps other S-nitrosylated proteins, play a role in resisting ROS and RNS damage. Consequently, the up-regulation of thioredoxin interacting partner proteins is expected to protect cells from ROS and RNS damage in a region populated by M1 macrophages or neutrophils which are absent from inflammation in BPH. Given this possibility, we sought to distinguish BPH from prostate cancer based on the prevalence of thioredoxin interacting protein partners in the adjacent stromal tissue.

Since taking the measure of the possible up-regulation of the full spectrum of thioredoxin interacting protein partners with immunochemistry using antibodies to known proteins would be both difficult and time consuming, in this report we sought to distinguish between these two forms of inflammation by using nanodevice-borne bacterial thioredoxin ligands [9]. Bacterial thioredoxin is a structural homolog of human thioredoxin and is an effective substrate for human thioredoxin reductase [10]. It is expected to bind to any thioredoxin interacting protein partner in general and to proteins essential to protection against reactive oxygen and reactive nitrogen species like H_2_O_2_ and NO in particular (e.g. thioredoxin reductase 1 (TXNRD1), thioredoxin reductase 2 (TXNRD2) [7] and thioredoxin peroxiredoxins like PRDX [11], [12]). Our rationale was that if a macrophage and neutrophil-based inflammation in the tumor microenvironment produces reactive oxygen and reactive nitrogen species, protective thioredoxin protein partners must be upregulated in order to maintain stromal cell viability, whereas this would be unnecessary in the CD4+ T-Lymphocyte-based inflammation seen in BPH.

## Materials and Methods

### Patients

Frozen prostate tissue specimens from 35 patients, who underwent surgical resection for prostate cancer at the City of Hope, were obtained as discard tissue under a City of Hope Institutional Review Board (IRB) approved protocol. Written informed consent was obtained from all participants before inclusion in this study. Cases were staged according to the TNM staging of prostate cancer and tumors were graded on the Gleason's scale as standard clinical care prior to release for experimentation. Table 1 lists the clinicopathologic characteristics of the patients obtained after surgery.

**Table 1:** 

	All Patients	BPH*	PCA
Number of Patients	35	17	18
Age At Prostatectomy (Std)	65.3 (7.2)	65.1 (7.2)	65.4 (7.3)
Gleason Sum, N (%)			
<7	13 (40.0%)	10 (58.8%)	3 (16.6%)
7	13 (37.2%)	7 (41.2%)	6 (33.3%)
>7	9 (25.7%)	0	9 (50.0)%)
ND-Trx_3_ Score, N (%)			
0 to 1	19 (54.3%)	12 (70.6%)	7 (38.9%)
2 to 3	16 (45.7%)	5 (29.4%)	11 (61.1%)
Gleason's Grade (major)	3.33 (0.526)	3.2 (0.41)	3.5 (0.62)
Gleason's Grade (minor)	3.75 (0.869)	3.2 (0.65)	4.4 (0.85)
Gleason's Sum	7.07 (1.25)	6.4 (0.63)	7.9 (1.39)
Extra Capsular Extension	8:35	2:17	6:18
Lymph Node Invasion	2:35	0:17	2:18
Seminal Vesicle Invasion	4:35	1:17	3:18
Positive Margin	7:18	0:17	7:18
% Tumor Volume	19.8 (16.2)	13.4 (10.2)	26.8 (21.6)
Total Tumor Volume	63.3 (43.5)	70.1 (59.8)	52.5 (14.0)
T0	2	2	0
T2a	8	5	3
T2b	4	3	1
T2c	14	6	8
T3a	2	0	2
T3b	3	1	2
T4	2	0	2

* Prostate cancer was detected in each specimen following surgery, however serial frozen sections were found to either lack a lesion (BPH:Benign Prostatic Hyperplasia) or contain a lesion (PCA: Prostate Cancer).

### Reagents

Mouse monoclonal antibodies to thioredoxin reductase 1, TXNRD1 (HPA 001395, Sigma-Aldrich), TXNRD2 (HPA 003323, Sigma-Aldrich) and PRDX1 antibody (ab59538, Abcam) were obtained from Sigma-Aldrich (St. Louis, MO) or Abcam (Cambridge, MA), respectively. Avidin-biotin-peroxidase complex (ABC), biotinylated horse antimouse immunoglobulins and normal horse serum were purchased from Vector Laboratories, Inc. (Burlington, CA).

Unmodified phosphoramidite monomers, with either standard or mild protecting groups, along with DNA solid supports and other reagents were purchased from Sigma-Proligo (St. Louis, MO) and Applied Biosystems, (Foster City, CA). TMP-FdU-CE Phosphoramidite for 5-5 fluoro-deoxycytosine insertion was from (Glen Research, sterling, VA) as was 5′-Fluorescein-dT Phosphoramidite used to fluorescently label DNA.

All other reagents used were of the highest purity available from various commecial sources (e.g. Sigma-Aldrich Chemical Co., St. Louis, MO).

### Clinical and Laboratory Assessment

Sets of adjacent sections were obtained from each patient specimen. Adjacent frozen sections (5 µm-thick) from resected prostate specimens were stained with Hematoxylin and Eosin (H&E) to determine whether or not prostate cancer was present in the adjacent section set or not. Although each patient in this study showed evidence of prostate cancer as determined by post surgical pathology, seventeen section sets lacked any evidence of prostate cancer, with Benign Prostatic Hyperplasia the only demonstrable pathological feature. Eighteen section sets contained prostate cancer. In those sets the region containing prostate cancer, identified in H&E stained slide, was marked by the pathologist, who was in turn blinded to the fluorescence study. Adjacent sections from selected specimens were stained with Masson's Trichrome to identify reactive stromal regions [13], [14] and immunohistochemically stained with TXNRD1, TXNRD2 or PRDX1 antibody as described [3]. Slides were photographed using an AX70 automated upright microscope. Immunohistochemical reagents were from DAKO Inc. Carpinteria CA.

### Nanodevice Assembly

**Figure 1:**
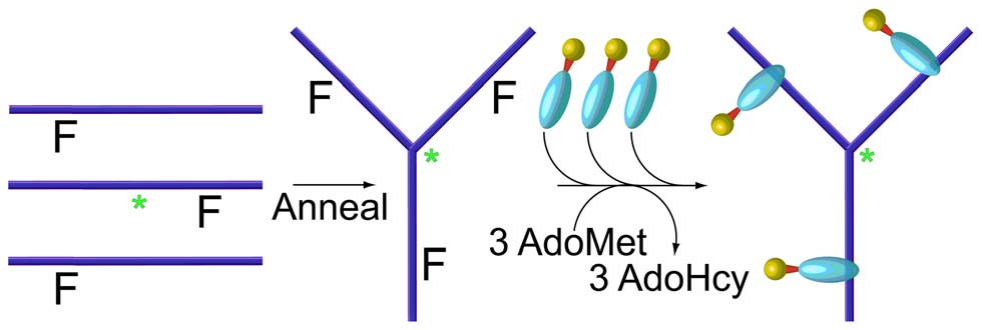
Schematic Diagram of the Self-Assembly Sequence for the Nanodevice (NP-Trx_3_). The fluorescein-labeled nanodevice (ND-Trx_3_) displays three copies of the bacterial thioredoxin (Trx) as a cellular targeting ligand. It is assembled by annealing three synthetic oligodeoxynucleotides containing fluoresein (*) at a centrally-located site and 5-Fluorocytosine (F) at each of the three methyltransferase recognition sites, followed by covalent linkage of methyltransferase fusion proteins as previously described [19]. Fluorescence labeling permits visualization of the bound device with fluorescence microscopy. Multivalency improves the avidity of the device since many of the known thioredoxin interacting partners (*e.g.* the human thioredoxin reductases) are dimeric [9].

The nanodevice (ND-Trx_3_) is a self-assembled molecular device, which targets thioredoxin-binding proteins. It consists of a DNA scaffold, which displays three copies of the thioredoxin redox protein for targeting, and a fluorescein label used for detection in fluorescence microscopy (Figure 1). Methods used for DNA synthesis have been described [15], [16]. The methods used to assemble and analyze the ND-Trx3 (Figure 1) have been described [17], [18]. Briefly, a DNA scaffold was assembled from synthetic oligodeoxynucleotides carrying 5-fluorocytosine, as a trapping agent for methyltransferase fusions, and fluorescein for detection by fluorescence microscopy. Once the DNA scaffold was assembled, a covalent complex is formed between the thioredoxin-M•*Eco*RII DNA methyltransferase fusion protein and the second 5-fluorocytosine in the C^F^CWGG sequence recognized by the DNA methyltransferase in the DNA scaffold. Assembly of the device was monitored by microfluidics-based gel retardation [18].

### Nanodevice Binding and Assessment

The methods used for the binding of the nanodevice to the sections of frozen prostate tissues were as previously described [17]. Briefly, frozen tissue sections (5 µm-thick), containing PCA or BPH were placed on histologic glass slides and incubated for 1 minute with 20 nM of fluorescence-labeled ND-Trx_3_ in 200 µl of ice-cold PBS or with 200 µl of PBS alone which functioned as a negative control. Each tissue section was then washed with 200 µl of ice-cold PBS, fixed and sealed for micriscopic examination. The slides were analyzed by fluorescent microscopy as previously described [17]. A tiled image photographed at 100X magnification allowed visualization of the entire tissue section to identify regions of ND-Trx_3_ binding.

The binding of the nanodevice to the tissue samples was evaluated using a method similar to that described in Ayala et al 14]. Staining around the edges and folds and staining due to calcium deposits within the prostate tissue were not included in the analysis. For each patient specimen, a tiled image of an entire prostate tissue section was examined. As noted below, binding fluorescence was largely confined to the stromal component of each tissue section. To evaluate the level of nanodevice binding, fluorescence was graded on a scale of 0–3. 0 =  no fluorescent staining, 1 =  mild staining with one localized area of staining, 2 =  moderate fluorescence with two to three discontinuous areas of staining, 3 =  high levels of fluorescence with broad areas continuous fluorescence staining. Two investigators who were blinded to the histological diagnoses evaluated the intensity of the nanodevice binding. Prior to evaluating specimens each blinded investigator was shown a tiled fluorescence image not used in the analysis that showed: a tissue section folding artifacts, punctate calcium deposit fluorescence and regions of localized and broad continuous fluorescence so that evaluation could be standardized between investigators. The average of the two numerical grades assigned by each investigator was used in the statistical analysis.

### Staining

Serial sections (5 µm-thick) of PCA or BPH tissue specimens were subjected to immunostaining. Briefly, sections were fixed in cold acetone for 10 minutes to air dry then blocked with Protein Block from DAKO, Inc. Carpinteria, CA. Slides were then incubated with primary antibody at room temperature at the following concentrations and times respectively: Anti-TXNRD2 (2 µg/ml) overnight, Anti-TXNRD1 (0.4 µg/ml) overnight, Anti-Peroxiredoxin 1 (2.5 µg/ml) 30 minutes. Each slide was subsequently washed in DAKO Buffer and incubated in secondary antibody (Rabbit/Mouse polymer (DAKO, Inc. Carpinteria, CA) for 30 minutes at room temp. After washes in DAKO buffer, slides were incubated with the chromogen diaminobenzidine tetrahydrochloride (DAB), counterstained with hematoxylin, and mounted. Adjacent sections were also stained with Hematoxylin and Eosin or Masson's Trichrome by standard techniques.

### Microscopic Analysis

Tiled images photographed at 100× magnification were created from each of three slides: one stained with H&E, one with Trichrome and one immunostained with TXNRD1, TXNRD2, and peroxirdoxin as previously described [9]. To identify the regions of the stroma verses the cancer, tiled images of adjacent slices of selected cases stained with H&E, Masson's Trichrome and immunohistochmically stained with TNXRD2 and actin were displayed sided by side.

### Statistical Analysis

Differences in binding levels were tested between BPH and PCA using the t-test. We first tested for variance heterogeneity and concluded that the pooled variance test was appropriate. The test for unequal variance was not rejected at 0.05 (p>0.8 with the Brown-Forsythe test).

## Results

### Evaluation of Binding of Nanodevice in Prostate Cancer or BPH

**Figure 2:**
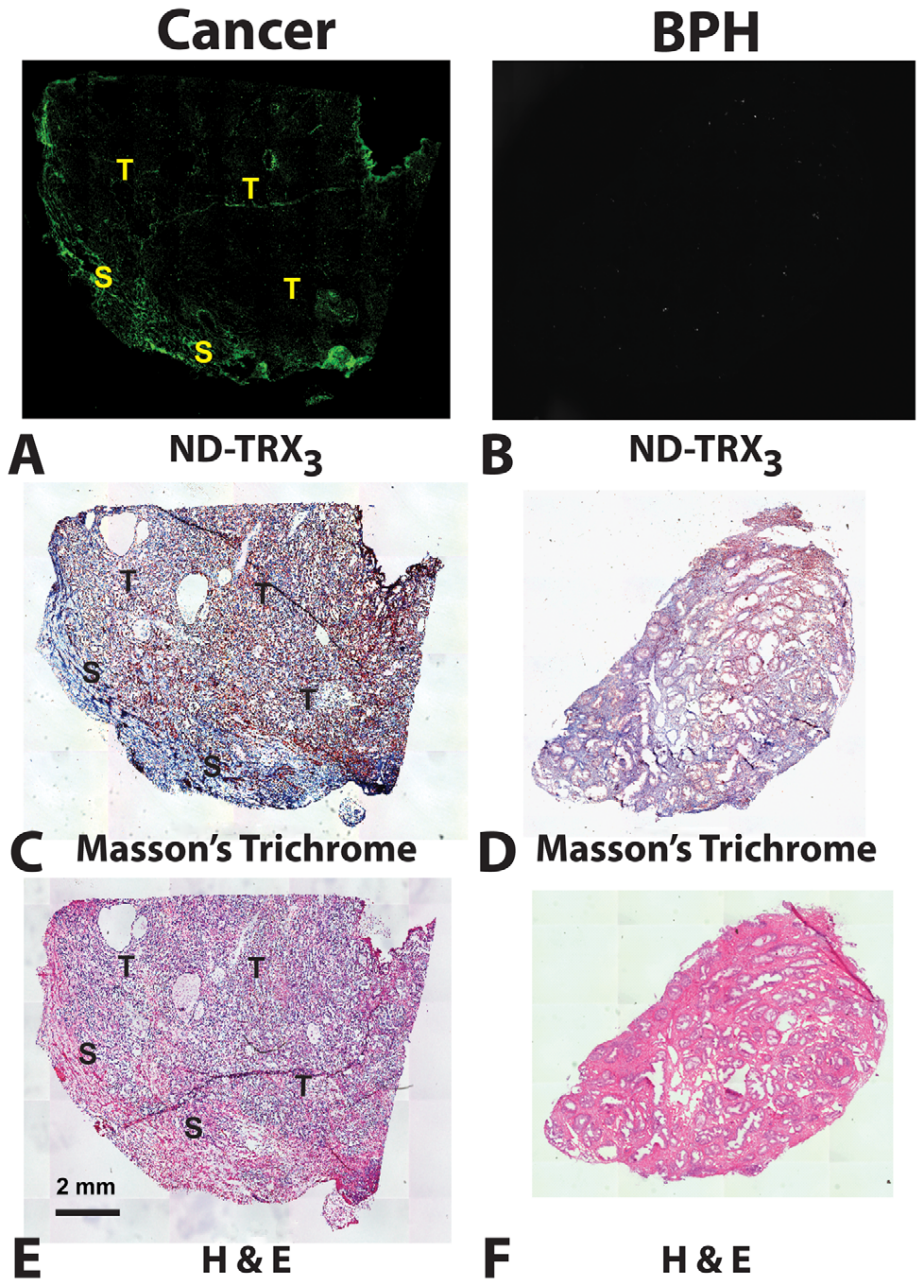
ND-Trx_3_ Binding to PCA and BPH in Frozen Tissue Sections. Representative frozen tissue sections were incubated for 1 minute with 20nM of the fluorescence-labeled ND-Trx_3_ in 200 µl of ice cold PBS or with 200 µl of PBS alone as control. Adjacent sections were also stained with H&E and Masson's Trichrome. The slides were then analyzed by fluorescent microscopy and a tiled image of the entire tissue slice was obtained to identify regions of ND-Trx_3_ binding. Tiled images photographed at 100X magnification allowed visualization of the entire tumor specimen after size reduction. Tumor (T) and Stromal (S) regions are indicated in each panel. PBS: Phosphate Buffered Saline, H&E: Hematoxylin and Eosin. Trichrome-stained sections were counter stained with hematoxylin (blue nuclear stain). The pattern of fluorescence observed with the nanodevice in the cancer specimen (**A**) was confined to the stromal region identified as the violet region in the adjacent section stained with Masson's Trichrome (**C**). Control BPH sections (**B**) did not bind the nanodevice significantly and yielded very low levels of fluorescence, and only weak staining with Masson's Trichrome (**D**). H&E stained sections containing tumor gave blue color in the tumor regions and light brown staining in regions of reactive stroma (**E**) and with BPH (**F**).

Staining of adjacent serial sections of tissue specimens of PCA or BPH with H&E allowed visualization of many details of cellular components (Figure 2). Each case was referenced to a positive fluorescence control using the same parameters for the image acquisition, and post-processing. The intensity of the nanodevice binding to the stroma was evaluated by two independent investigators and the averaged values were used for the subsequent analysis.

The nanodevice (ND-Trx_3_) was found to bind, as indicated by the intensity of fluorescence activity, to the regions of stroma, which were located adjacent to tumor lesions. In 11 of 18 cases of PCA, the intensity of ND-Trx_3_ binding was scored greater than 1. Regions that exhibited binding activity with the nanodevice (Fig. 2A: green fluorescence) were also positive for the staining with Masson's trichrome (Fig. 2C: violet staining). The binding activity of the nanodevice to the epithelial cells as well as the stroma of BPH was undetectable (Fig. 2B: absence of green fluorescence) as were the staining with Masson's trichrome (Fig. 2D: absence of blue staining).

### Immunohistochemical analysis of thioredoxin binding proteins

**Figure 3:**
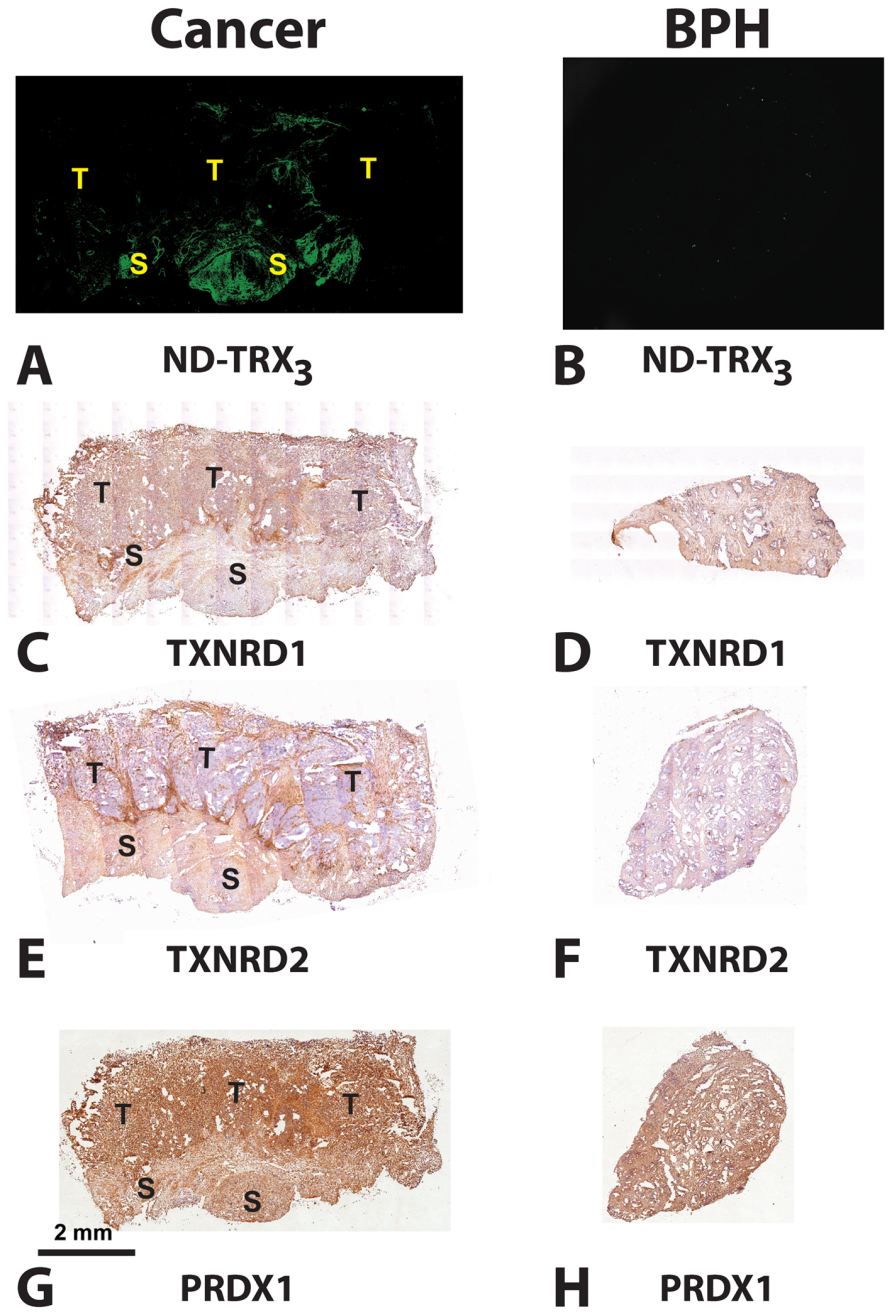
Comparison of the Pattern of Nanodevice Binding with that of Immunohistochemical Staining with Antibodies to TXNRD1, TXNRD2 and PRDX1 in Representative Tumor Specimens. Adjacent sections (5 µm-thick) of resected tissue specimens containing prostate cancer or BPH were incubated with ND-Trx_3_, or immunohistochemically (IHC) stained with antibodies to TXNRD1, TXNRD2, or Peroxiredoxin. Each immunohistochmically stained section was also counterstained with hematoxylin (blue nuclear staining). The fluorescence pattern observed with the nanodevice (**A**) was similar to the brown staining in the stromal regions (S) and at the nterface between stroma (S) and tumor (T) observed TXNRD2 (**E**). Fluorescence due to the nanodevice was essentially absent from BPH specimens (**B**). This pattern was similar to that of antibody to TXNRD2, which did not effecively stain BPH (**F**). Anti-TXNRD1 stained the cancer regions (T) more effectively than the stromal regions (S) (**C**). Anti-TXNRD1 gave only light brown staining with BPH (**D**). Antibody to PRDX1 gave strong brown staining in the cancer (T) with somewhat less staining in the reactive stroma (S) (**G**). Anti-PRDX1 gave uniform brown staining with BPH (**H**).

A sufficient number of unstained consecutive tissue sections were available for immunohistochemical analysis, in five cases where PCA was present in the sections obtained and in five cases where only BPH was present in the sections obtained. These sections were stained with antibodies to TXNRD1, TXNRD2 or PRDX1 to determine if any of them mirrored the fluorescence signal obtained with the nanodevice. The anti TXNDR1 or PRDX1 antibody showed strong reactivity with the tumor cells (Fig. 3C and 3G: brown color staining) and weak or strong reactivity, respectively, with the stroma (Fig. 3C: brown color staining), which was located adjacent to tumor lesions in the representative cases of PCA. The patterns of reactivity of antibody to either TXNRD1 or PRDX1 showed no overlap with that of the fluorescence of the thioredoxin-targeted nanodevice (Fig. 3A, C, G).

In contrast, anti-TXNRD2 antibody exhibited little or no staining of the tumor cells (Fig. 3E: absence of brown color staining indicated by open arrows), whereas stroma located adjacent to tumor lesions showed strong staining (Fig. 3E: strong brown color staining). Staining was particularly strong at the interface between the stroma and tumor. Overall, the pattern of reactivity of anti-TXNRD2 antibody roughly overlapped with that of the fluorescence of the nanodevice (Fig. 3A, E).

**Figure 4:**
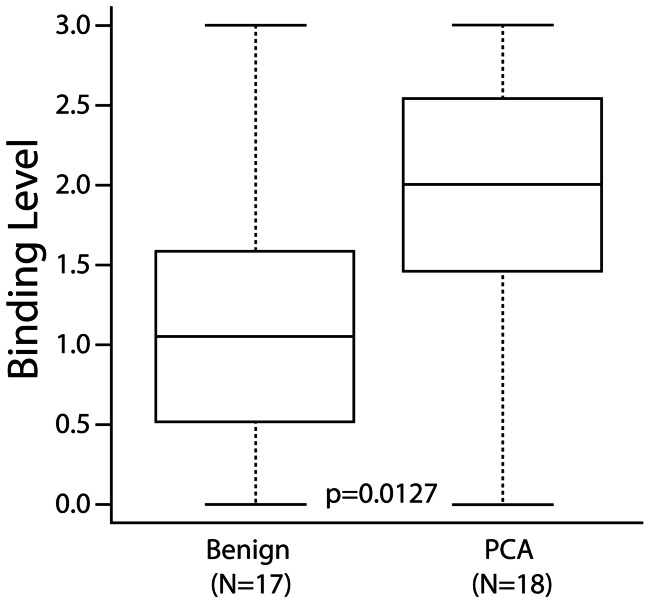
Distinguishing PCA from BPH Based on Nanodevice Binding to Thioredoxin Interacting Proteins. Surgically resected tissue specimens were obtained from 35 patients. Serial sections of the tissue specimens (5 µm thick) were incubated with 20 nM of the nanodevice in PBS and 1% BSA. Fluorescence binding was observed and a numerical grading system was given as the level of nanodevice fluorescence in the stroma by two independent investigators (the author EMS and GB from acknowledgements). **0-** No visible signal, **1-** weakly visible signal, **2-** a moderately intense visible signal and **3-** a bright and intense signal within the reactive stroma. The binding level for PCA-associated stroma was significantly greater than that of BPH (p = 0.0127) based on the t-test.

The strong correlation between fluorescence associated with thioredoxin interacting protein partners in PCA adjacent stroma is quantified by the data given in Fig. 4. The intensity of nanodevice binding to the stroma (Figure 4) was significantly associated with PCA as compared to that of BPH (p = 0.0127). The statistics used in boxplots were the means with 95% confidence intervals for the hinges and extrema for the whiskers. False positive and false negative outliers may be reactive stroma or cancer above or below the stained section. Even so, the conditional power of the current sample size (N = 35) is 91%. However, it is important to point out that the results given in Figure 4 show only a moderate correlation with tumor stage given in Table 1. While the intensity of nanodevice binding to the stroma appears to be correlated with a number of patient parameters given in Table 1 (e.g. % tumor involvement, the presence of positive surgical margin and Gleason sum), the random sections collected from each specimen do not fully characterize the specimen.

## Discussion

Although inflammation appears to play a crucial role in both PCA [1] and BPH [2], our results support the observation that the inflammation associated with BPH consists largely of IL-15 and γ-interferon recruited CD4+ T-Lymphocytes [2], and it contrasts with the tumor-associated macrophages present in the tumor microenvironment. Our results also suggest that the M2-like phenotype [1] postulated to promote tumor angiogenesis and metastasis [1] involves the up-regulation or extends the half-life of thioredoxin interacting protein partners in the tumor adjacent stroma.

Since thioredoxin expression appears to be a link between ROS and RNS stress due to its ability to act as a chemoattractant for neutrophils, monocytes and T-cells [6], the presence of thioredoxin interacting protein partners in the tumor adjacent stroma detected by the thioredoxin-targeted nanodevice appears to indicate ongoing ROS and RNS stress and inflammation in fluorescent regions of the tissue sections. Moreover, the high levels of TXNRD2 expression seen at the interface between the stroma and the tumor (Figure 3) are consistent with an ongoing wound response in tumor-associated stroma. Since TXNRD2 reduces H_2_O_2_ to H_2_O [7], its up-regulation is expected to protect stromal cells from ROS damage in a region populated by M1 macrophages or neutrophils which are absent from inflammation in BPH.

The data presented in Figure 4 suggests that when a macrophage and neutrophil-based inflammation is generated to produce ROS and RNS, protective thioredoxin systems must be upregulated to maintain cellular viability. This pathway appears to be unnecessary in BPH because the CD4+ T-Lymphocyte-based inflammation does not generate a respiratory burst that would adversely affect cellular viability. The intensity of nanodevice binding to the stroma was significantly associated with PCA as compared to that of BPH (p = 0.0127), providing strong support for the hypothesis that an ROS producing inflammatory response is largely absent from BPH. The binding activity of the nanodevice to the epithelial cells as well as the stroma of BPH was undetectable suggesting that the targeted thioredoxin proteins are not present in nonreactive stroma, and that the nanodevice is specifically binding to reactive stroma present in prostate cancer.

As noted in results, the intensity of nanodevice binding to the stroma is weakly associated with a number of patient parameters given in Table 1 (e.g. % tumor involvement, the presence of positive surgical margin and Gleason sum), however, the random sections collected from each specimen do not fully characterize the specimen. All specimens were from proven cases of prostate cancer even though many sections were found to be devoid of prostate carcinoma. Consequently, these correlations appear to result from the increased likelihood that a series of adjacent sections taken at random depth from a resected prostate gland are more likely to contain tumor in cases involving high tumor volume, positive surgical margins, extracapsular extension and or seminal vesicle involvement.

## Conclusions

Our data suggests that nanotechnology can be used for the discovery of new biomarkers of reactive stroma in prostate cancer. In addition, the nanodevice has the advantage of binding to a broad range of thioredoxin interacting protein partners, which distinguish PCA associated stroma from that associated with benign tissue regions based on the inflammatory response and may not be seen using antibodies against any specific thioredoxin binding protein expressed in the stroma. Although the subjective qualitative analysis of the nanodevice binding may introduce bias, the data obtained in this study are significant even for the small sample size reported here. Despite this limitation, the statistical analysis of the data, coupled with the detection of the reactive stroma using Masson's Trichrome stain and the expression levels of the thioredoxin reductases support the hypothesis that thioredoxin and its thioredoxin interacting protein partners play an important role in prostate cancer.

The data presented here also clearly demonstrated that the thioredoxin-targeted nanodevice can be used in a functional assay to identify reactive stroma in prostate cancer, providing strong support for the hypothesis that an ROS and/or RNS producing inflammatory response is associated with PCA and distinct from BPH. In addition, these results suggest that reactive stroma may be an effective target for prostate cancer therapeutics.
